# Occlusion dysfunction and Alzheimer’s disease: Mendelian randomization study

**DOI:** 10.3389/fnagi.2024.1423322

**Published:** 2024-07-05

**Authors:** Qing Wang, Wenyu Zhen, Rui Hu, Zifei Wang, Yuqiang Sun, Wansu Sun, Chunxia Huang, Jianguang Xu, Hengguo Zhang

**Affiliations:** ^1^Key Laboratory of Oral Diseases Research of Anhui Province, College and Hospital of Stomatology, Anhui Medical University, Hefei, China; ^2^Department of Anesthesiology and Perioperative Medicine, The Second Affiliated Hospital of Anhui Medical University, Hefei, China; ^3^Department of Stomatology, Anhui Public Health Clinical Center, The First Affiliated Hospital of Anhui Medical University, Hefei, China

**Keywords:** occlusion dysfunction, Alzheimer’s disease, periodontal disease, Mendelian randomization, epidemiology

## Abstract

**Aim:**

Occlusion dysfunction (OD) is increasingly linked to Alzheimer’s disease (AD). This study aimed to elucidate the causal relationship between OD and AD using Mendelian randomization (MR) analysis.

**Materials and methods:**

Genome-wide association study (GWAS) meta-analysis data obtained from FinnGen, IEU Open GWAS, and UK Biobank (UKBB) was represented as instrumental variables. We validated the causal relationship between periodontal disease (PD), loose teeth (PD & occlusion dysfunction), dentures restoration (occlusion recovery), and AD.

**Results:**

According to the MR analysis, PD and AD have no direct causal relationship (P = 0.395, IVW). However, loose teeth significantly increased the risk of AD progression (P = 0.017, IVW, OR = 187.3567, 95%CI = 2.54E+00−1.38E+04). These findings were further supported by the negative causal relationship between dentures restoration and AD (P = 0.015, IVW, OR = 0.0234, 95%CI = 1.13E-03−0.485).

**Conclusion:**

The occlusion dysfunction can ultimately induce Alzheimer’s disease. Occlusion function was a potentially protective factor for maintaining neurological health.

## Introduction

Occlusion dysfunction (OD) commonly arises from missing teeth and severe periodontal disease (PD) (Ramseier et al., 2017). As a chronic inflammatory disease, PD is widespread among the elderly and has become a crucial global health issue (Eke et al., 2015). Mechanistically, PD and related complications were closely associated with neurodegenerative diseases, such as Alzheimer’s disease (AD) (Hajishengallis, 2022). In which, OD serve as the risk factor for the maintenance of neurological health (Teixeira et al., 2014). Based on the importance of neurological health, the potential causal relationships among OD, PD, and AD deserve in-depth exploration.

As a chronic neurodegenerative disorder with complex etiology, AD is characterized by the accumulation of a protein called amyloid-β (Aβ) in brains. During autopsies, PD related *Porphyromonas gingivalis* (Pg) have been found in the brains of AD patients (Jungbauer et al., 2022). These bacteria can invade the central nervous system and cause inflammation by producing certain molecules (Singhrao et al., 2015). Furthermore, the elevated levels of inflammatory factors resulting from PD can contribute to an inflammatory in the brain (Kamer et al., 2008a,b). Meanwhile, AD patients often experience severe PD due to a combination of factors, including the diminished ability and lack of motivation to maintain oral hygiene (Gao et al., 2020). Currently, there is a lack of direct clinical evidence demonstrating the clear association between AD and PD.

Previous research has demonstrated that OD can contribute to brain impairments and cognitive decline, such as reduced synapses, degeneration of nerve cells, and inhibition of neurotransmitter release (Terasawa et al., 2002; Okihara et al., 2014). Additionally, AD is often accompanied by atrophy in both the cortical and subcortical areas of the brain, particularly in the internal olfactory cortex and hippocampus (Teipel et al., 2006; Wang et al., 2020). Thus, occlusal function has emerged as a novel factor in understanding the causes of AD.

Mendelian randomization (MR) is a novel methodology to assess causal relationships between target exposures and diseases or traits. While randomized controlled trials (RCTs) are considered the gold standard approach for testing causality, they are often constrained by financial and ethical considerations, and confounding factors may introduce biases (Sekula et al., 2016). MR helps overcome these issues by using genetic variants, known as single nucleotide polymorphisms (SNPs), which are strongly associated with the traits being studied. This approach helps reduce biases that can occur in traditional RCTs (Ebrahim and Davey Smith, 2008). MR research has yielded significant findings regarding the potential causal associations between environmental risk factors and diseases, with numerous high-impact publications in esteemed journals (Ference et al., 2019; Jones et al., 2021).

In this study, we aimed to systematically evaluate the causal association between PD, OD (loose teeth), occlusion recovery (dentures restoration), and AD using MR analysis. Significantly, our findings demonstrate a strong causal relationship between OD and AD (*p* = 0.0171, IVW), providing a novel perspective on the impact of PD on AD and highlighting the crucial role of OD in the development of AD. This article contributes to the development of new therapeutic strategies for the prevention and treatment of AD.

## Material and methods

**FIGURE 1:**
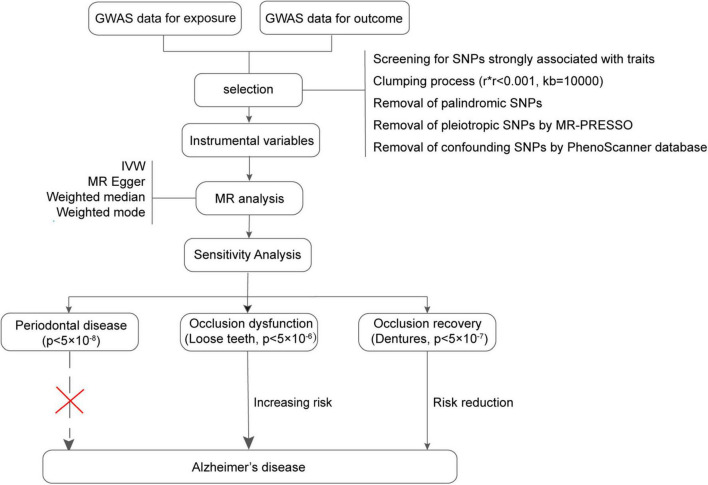
Flowchart of the Mendelian randomization process in this study. GWAS, Genome-wide association study; MR, Mendelian randomization.

The datasets for exposure, including PD, loose teeth, and dentures restoration, as well as the outcome data for AD, were systematically searched from multiple genome-wide association study (GWAS) meta-analysis data sources, including IEU Open GWAS, FinnGen, and UK Biobank. Initially, all combinations of conditions were screened using MR analysis. The datasets with the most significant outcomes were selected for further investigation (Figure 1). It is important to note that all the data used in this study are publicly available (Table 1), and the download link is provided in the Data Availability Statement section.

**TABLE 1:** Detailed information on GWAS summary data.

Trait	Sample size	Population	Sex	Attribute	GWAS ID/Cohort
Periodontal Disease-related phenotype (Socransky)	975	European	mix	exposure	ebi-a-GCST003484
Occlusion Dysfunction (Loose Teeth)	461113	European	mix	exposure	ukb-b-12849
Occlusion Recovery (Dentures restoration)	498812	European	mix	exposure	UKB
Alzheimer’s Disease (Late onset)	54162	European	mix	outcome	ebi-a-GCST002245
Alzheimer’s Disease (Early onset)	171743	European	mix	outcome	FinnGen

The GWAS IDs provided in the table can be accessed on the IEU OpenGWAS website. UKB, UK Biobank.

### Instrumental variables

According to the previous study (Emdin et al., 2017), instrumental variables must satisfy three key assumptions: relevance, independence, and exclusion restriction. To address the requirements of MR, we have outlined the steps for screening instrumental variables. The screening criteria are as follows: (1) Initially, we selected SNPs that exhibited a strong association with the exposure (*p* < 5.0 × 10^–8^, PD; *p* < 5.0 × 10^–6^, loose teeth; *p* < 5.0 × 10^–7^, dentures restoration). (2) The linkage disequilibrium (LD) of these SNPs was calculated, and only those SNPs meeting the following conditions were retained: *r*^2^ < 0.001, kb = 10000 (3) To minimize bias, we eliminated all palindromic SNPs among the retained ones. (4) Using the R package “MR-PRESSO”, we identified and removed a pleiotropic among all reserved SNPs. (5) Additionally, we employed the PhenoScanner database^1^ to exclude confounding factors and risk variables (Table 2). The remaining SNPs were subsequently utilized for MR analysis (Table 3).

**TABLE 2:** SNPs associated with known confounders.

Exposure	Sort	SNP	confounding factor	Reference (PMID)
Loose teeth	1	rs1117062	Diastolic blood pressure	33766239
Loose teeth	2	rs312989	hypertension	33766239
Loose teeth	3	rs72720396	Alcohol usually taken with meals	32230811
Dentures restoration	1	rs11084095	loose teeth	NA
Dentures restoration	2	rs12521680	Seen doctor for nerves, anxiety, tension or depression	21537355
Dentures restoration	3	rs148158713	Smoking	24924665
Dentures restoration	4	rs1794514	Cancer	32382138
Dentures restoration	5	rs2046850	hypertension	33766239
Dentures restoration	6	rs362270	Alcohol intake frequency	32230811
Dentures restoration	7	rs4795386	Rheumatoid arthritis	36198219
Dentures restoration	8	rs55958997	Smoking	24924665
Dentures restoration	9	rs6058638	Weight	19358976
Dentures restoration	10	rs72982972	Body mass index	19358976
Dentures restoration	11	rs7620314	Body mass index	19358976

By using PMID, the relevant literature on confounders can be accessed on the PubMed website. SNP, single nucleotide polymorphism.

**TABLE 3:** Instrumental variables for MR analysis.

Exposure	Sort	SNP	EA	OA	Beta	SE	*P*-value
PD	1	rs1156327	C	T	−1.452	0.230	3.01E-10
PD	2	rs1633266	T	C	−0.932	0.168	3.09E-08
PD	3	rs17184007	C	T	1.346	0.232	6.86E-09
PD	4	rs17718700	C	T	1.217	0.223	4.58E-08
PD	5	rs3811273	G	A	1.216	0.203	2.06E-09
Loose teeth	1	rs11049359	C	T	0.003	0.0005	2.60E-08
Loose teeth	2	rs11220245	G	A	0.002	0.0005	2.70E-07
Loose teeth	3	rs279743	C	T	−0.002	0.0004	3.70E-06
Loose teeth	4	rs2947122	A	G	0.003	0.0006	1.20E-06
Loose teeth	5	rs34438171	T	C	0.003	0.0006	4.30E-06
Loose teeth	6	rs3763469	C	T	−0.003	0.0005	6.80E-07
Loose teeth	7	rs4801882	A	G	0.002	0.000	1.00E-07
Loose teeth	8	rs61823158	G	A	−0.004	0.0008	4.10E-07
Loose teeth	9	rs6586364	T	G	0.003	0.0007	1.80E-06
Loose teeth	10	rs7028167	C	A	0.002	0.0005	4.00E-06
Loose teeth	11	rs714962	G	A	0.002	0.0004	2.60E-06
Loose teeth	12	rs72664597	G	A	0.004	0.0008	4.80E-07
Loose teeth	13	rs982894	G	A	0.002	0.0004	4.20E-06
Dentures restoration	1	rs10048146	G	A	0.008	0.001	3.50E-13
Dentures restoration	2	rs10956340	C	A	−0.005	0.001	8.06E-08
Dentures restoration	3	rs10987017	G	A	0.005	0.001	8.28E-08
Dentures restoration	4	rs111659883	T	C	−0.005	0.001	3.55E-07
Dentures restoration	5	rs1122171	T	C	0.012	0.001	4.55E-44
Dentures restoration	6	rs117737827	T	C	0.007	0.001	1.61E-09
Dentures restoration	7	rs121908120	A	T	−0.022	0.003	3.54E-17
Dentures restoration	8	rs1482698	C	G	0.005	0.001	4.89E-10
Dentures restoration	9	rs2238651	T	C	0.005	0.001	2.61E-07
Dentures restoration	10	rs2270764	G	A	0.008	0.001	7.80E-20
Dentures restoration	11	rs2421616	G	A	−0.004	0.001	4.75E-07
Dentures restoration	12	rs2514310	G	A	−0.005	0.001	2.43E-07
Dentures restoration	13	rs4233366	T	C	0.005	0.001	4.52E-07
Dentures restoration	14	rs4445705	T	A	−0.007	0.001	1.65E-07
Dentures restoration	15	rs62254667	G	A	0.030	0.005	2.03E-08
Dentures restoration	16	rs72694438	A	G	0.006	0.001	1.39E-08
Dentures restoration	17	rs7367207	T	C	0.006	0.001	1.01E-10
Dentures restoration	18	rs77083638	G	A	0.007	0.001	2.18E-07
Dentures restoration	19	rs7864794	T	G	0.007	0.001	7.26E-08
Dentures restoration	20	rs924394	A	G	−0.006	0.001	1.86E-09
Dentures restoration	21	rs933292	A	G	0.006	0.001	2.21E-07
Dentures restoration	22	rs9831002	G	T	0.005	0.001	9.96E-11

PD, Periodontal Disease; EA, effect_allele; OA, other_allele; SE, standard error.

### Mendelian randomization

To investigate the causal relationship between exposure and outcome, we employed four methodologies: MR Egger, Weighted median, Inverse variance weighted (IVW), and Weighted mode. Previous research has demonstrated that IVW analysis is the most reliable and accurate approach (Bowden et al., 2016). Therefore, we primarily relied on the IVW analysis, considering the additional methods as supplementary. When the p-value of IVW analysis was less than 0.05 and the odds ratio (OR) values exceeded 1 for all four methods, we inferred that exposure was a significant risk factor for AD.

### Sensitivity analyses

To ensure the reliability of our findings from the MR analysis, a sensitivity analysis was performed. Heterogeneity was assessed using the Cochrane Q test for both MR Egger and IVW methodologies. A Q_pval value greater than 0.1 indicates the absence of heterogeneity among the studies. Furthermore, we employed MR Egger to examine pleiotropy. An Egger_intercept close to 0 or a p-value (between the intercept and 0) greater than 0.05 suggests the absence of pleiotropy in the results. Additionally, we conducted a leave-one-out permutation analysis to evaluate the impact of individual SNPs on the overall results.

## Results

### Periodontal diseases

Based on our screening criteria, we identified 5 SNPs after excluding 1 palindromic SNP. Among these 5 SNPs, no palindromic SNPs, outlier SNPs, or confounding SNPs were detected. Our MR analysis revealed no causal association between PD and AD, as indicated by the MR results (*P* = 0.3950, IVW, Table 4).

**TABLE 4:** MR estimates for the association between exposures and outcome.

Exposure	MR method	No. of SNP	OR	95% CI	*P*-value	SE
PD	MR Egger	5	1.083	0.757 - 1.550	0.691	0.183
PD	Weighted median	5	0.979	0.913 - 1.049	0.538	0.035
PD	IVW	5	0.977	0.927 - 1.030	0.395	0.027
PD	Weighted mode	5	0.986	0.900 - 1.081	0.778	0.047
Loose teeth	MR Egger	13	331.197	4.29E-07 - 2.56E+11	0.590	10.442
Loose teeth	Weighted median	13	747.463	2.08E+00 - 2.68E+05	0.028	3.001
Loose teeth	IVW	13	187.357	2.54E+00 - 1.38E+04	0.017	2.194
Loose teeth	Weighted mode	13	3978.370	1.19E-01 - 1.33E+08	0.145	5.317
Dentures restoration	MR Egger	22	0.135	1.52E-05 - 1.19E+03	0.670	4.638
Dentures restoration	Weighted median	22	0.024	2.33E-04 - 2.566	0.118	2.375
Dentures restoration	IVW	22	0.023	1.13E-03 - 0.485	0.015	1.547
Dentures restoration	Weighted mode	22	0.018	6.90E-05 - 4.912	0.176	2.850

IVW, Inverse-Variance Weighted; OR, odds ratio; SE, standard error; CI, confidence interval.

### Loose teeth

**FIGURE 2:**
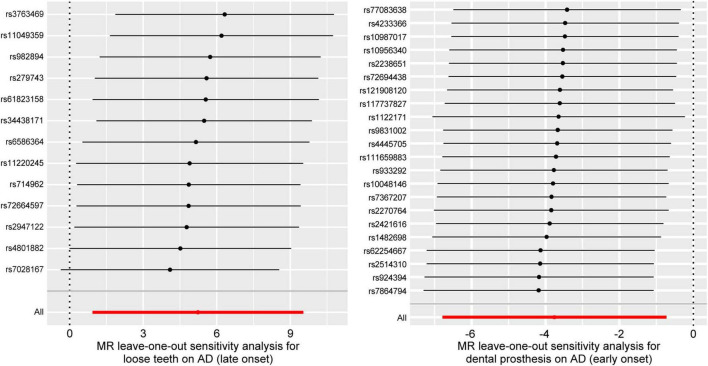
Forest plots for leave-one-out analysis in sensitivity analyses. MR analysis after removing SNPs one by one. AD, Alzheimer’s disease.

After applying screening criteria, we obtained a set of 13 instrumental variables (SNPs). This was achieved by removing 2 palindromic SNPs and 3 SNPs that were associated with confounding factors. Our MR-PRESSO and leave-one-out analyses did not identify any outlier SNPs (Figure 2).

**FIGURE 3:**
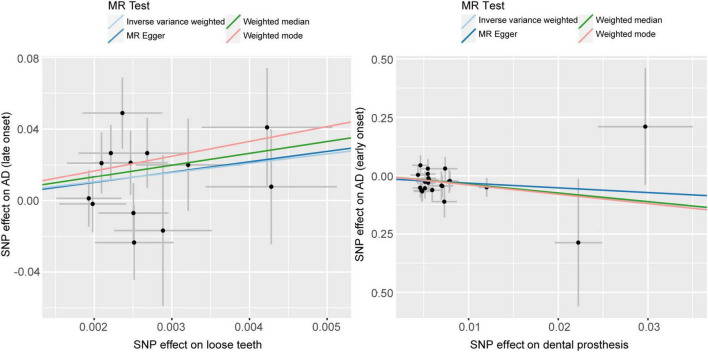
Scatter plots of causality. Perform linear regression on the results of the four different methods in MR analysis. The slope is equal to the beta. AD, Alzheimer’s disease.

Based on the results of the Weighted median method (*p* = 0.0275, OR = 747.4632, 95%CI = 2.08E+00 - 2.68E+05) and IVW method (*p* = 0.0171, OR = 187.3567, 95%CI = 2.54E+00−1.38E+04), we found a significant causal relationship between loose teeth and AD. Such a high OR (187.3567) implies a robust risk correlation between loose teeth and AD (Figure 3). To provide a comprehensive overview of the MR analysis, we have included the results of different methods in Table 4.

Furthermore, the Cochran’s Q test (Q_pval = 0.3774, MR Egger; Q_pval = 0.46, IVW) and MR-Egger (p [between intercept and 0] = 0.9564) did not reveal any evidence of heterogeneity or horizontal pleiotropy.

### Dentures restoration

Following the removal of 11 SNPs associated with confounding factors, we included a total of 22 SNPs for our MR analysis. Our analysis did not identify any SNPs as outliers (Figure 2).

Using the IVW analysis, we found a significant protective effect of dentures restoration against AD risk, with an OR of 0.0234 (95%CI = 1.13E-03−0.485) and a *p*-value of 0.0152 (Table 4, Figure 3).

We have also conducted tests to evaluate heterogeneity and horizontal pleiotropy. Both the IVW test (Q_pval = 0.8618) and the MR-Egger test (Q_pval = 0.8296, p [between intercept and 0] = 0.6932) did not provide evidence of heterogeneity or horizontal pleiotropy.

## Discussion

Recently, the prevalence of OD in AD patients and its significant impact on brain health have been increasingly recognized. This condition not only arises from the mobility and cognitive impairments in AD patients but also contributes to the characteristic neurodegeneration and brain damage associated with the disease (Nakamura et al., 2021). In this study, we conducted a systematic evaluation of the causal relationships between PD, OD, occlusion recovery, and AD using a two-sample MR analysis. Our study yielded three key findings. Firstly, PD does not directly cause AD. Secondly, OD is a risk factor for AD. Lastly, occlusion recovery can reduce the risk of AD. These findings are in line with previous studies in the field.

AD patients usually suffer from PD. Previous studies have consistently shown a link between PD and AD (Borsa et al., 2021). Pathogenic bacteria associated with PD can lead to brain nerve damage and cognitive impairment through their proteins and DNA (Chen et al., 2017; Long and Holtzman, 2019). However, due to the limitations of epidemiologic studies, such as confounding factors and reverse causation, evidence for a causal relationship between PD and AD remains limited (Noble et al., 2009). Previous studies cannot provide evidence to support PD as a risk factor for AD (Sun et al., 2020). Accordingly, we re-evaluated and validated this result by utilizing stronger correlated instrumental variables (*p* < 5.0 × 10^–8^). Meanwhile, oral dysfunction (including OD) may contribute to cognitive impairment, such as the progression of AD (Takahashi et al., 2023). As not a direct factor in AD development, PD can indirectly influence the disease by causing certain intermediate signs. Severe PD can result in tooth loosening and eventual loss, which are frequently associated with malocclusion in patients. Additionally, numerous studies have indicated the association between occlusal function and the maintenance of brain nerve health.

To verify the role of occlusal function in AD, we performed MR analysis utilizing GWAS summary data from the UKB on loose teeth as a proxy for OD. The results indicated a significant causal relationship between OD and AD, supporting our initial hypothesis.

Tooth loss correlates with the decline of cognition-related brain regions (Kobayashi et al., 2018). Occlusion dysfunction reduces sensory input from receptors around the teeth, subsequently resulting in degeneration of primary nerve cells involved in brain neurotransmission (Kubota et al., 1988). A study confirmed that tooth loss results in the denervation of nerve endings at the root apex of the apical trigeminal nucleus Vmes (Goto et al., 2020). Vmes is the only primary sensory neuron located within the central nervous system. Recent studies suggest that this alteration may permit the activation of inflammatory microglia, which, in turn, activate pathways involving pro-phosphorylating kinases and oxidative stress. This leads to tau hyperphosphorylation and aggregation, and consequent degeneration of the locus coeruleus (LC) located near Vmes (Matsumoto et al., 2023). Additionally, the LC is primarily responsible for the release of norepinephrine, which has been shown to have an inhibitory effect on inflammation. Therefore, the degradation of the LC enhances the inflammatory response, further increasing the number of inflammatory microglia and creating a vicious cycle. Additionally, other studies show that OD contributes to decreased expression of brain-derived neurotrophic factor (BDNF) (Takeda et al., 2016) and affects neurotransmitter release, such as the dopamine and acetylcholine in the hippocampus (Makiura et al., 2000; Kushida et al., 2008). As reduced brain volume and dysfunction are observed in AD patients (Teipel et al., 2006; Wang et al., 2020), OD can contribute to the development of AD through mechanisms such as Tau deposition, reduced neurotransmitter secretion, and decreased BDNF expression, all resulting from neurodegeneration. Additionally, it has been shown that chewing dysfunction may contribute to the development of AD by decreasing blood flow to the brain and affecting diet (Blazer, 2022).

As the effective therapeutic for occlusion recovery, dentures restoration significantly improved occlusal function in individuals with tooth loss (Campos et al., 2017). In addition, the MR analysis results indicate a significant reduction in the risk of AD associated with occlusion recovery, which is consistent with a controlled clinical trial (Okamoto, 2011). In which, improved occlusion function facilitates neurostimulation into the brain, promotes neurotransmitter transmission, and prevents the atrophy of cerebral nerves. Consequently, dental prostheses and occlusion recovery can slow down the progression of AD. This finding corroborates the idea that OD contributes to the progression of AD and emphasizes the importance of oral care or occlusal function for AD patients (Gao et al., 2020).

In this study, we utilized SNPs strongly associated with exposure and outcome to achieve randomization, bypassing the limitations of traditional RCTs. The heritability of exposure and outcome is crucial for determining if SNPs are robust proxies. Research indicates that AD (Loy et al., 2014; Quan et al., 2023) and PD (Loos and Van Dyke, 2020; Shaddox et al., 2021) are strongly hereditary, and loose teeth, a marker of advanced PD, have a clear genetic relationship (Morelli et al., 2020). Given the correlation between dentures restoration and occlusal function recovery, we use dentures restoration as a proxy. Occlusion function, influenced by genetic factors affecting teeth characteristics, can be compromised by genes increasing susceptibility to PD bacteria, leading to teeth loosening and dysfunction (Esberg et al., 2019). These genetic factors also affect the feasibility of occlusal function recovery, indicating a strong genetic correlation with dentures restoration.

While our study follows a rigorous logic and the results are cross-validated, there are limitations to be considered. The AD GWAS data obtained from the UK Biobank relied on self-reports, which may introduce inaccuracies due to self-cognitive biases. Nevertheless, a meta-analysis by Marioni et al. (2018) has suggested that self-reported AD can accurately represent a clinical diagnosis (Marioni et al., 2018). Additionally, as our study is based on a European population, the generalizability of our findings to other populations may be limited, and further validation in other races is needed in future research.

## Conclusion

Overall, our study provides important insights into the relationships between PD, OD, occlusion recovery, and AD. These findings contribute to a better understanding of the role of OD in AD development and emphasize the significance of occlusion recovery in the prevention of AD. Importantly, the preservation of optimal occlusion function represents a viable strategy to safeguard neurologic integrity.

## Data availability statement

The original contributions presented in the study are included in the article/supplementary material, further inquiries can be directed to the corresponding authors.

## Ethics statement

Ethical approval was not required for the study involving humans in accordance with the local legislation and institutional requirements. Written informed consent to participate in this study was not required from the participants or the participants’ legal guardians/next of kin in accordance with the national legislation and the institutional requirements.

## Author contributions

QW: Writing–review and editing, Writing–original draft, Formal analysis, Data curation. WZ: Writing–review and editing, Writing–original draft, Formal analysis, Data curation. RH: Writing–review and editing, Writing–original draft, Resources, Data curation. ZW: Writing–review and editing, Investigation, Formal analysis. YS: Writing–review and editing, Resources. WS: Writing–review and editing, Data curation. CH: Writing–review and editing, Supervision, Funding acquisition, Conceptualization. JX: Writing–review and editing, Supervision, Funding acquisition, Conceptualization. HZ: Writing–review and editing, Supervision, Funding acquisition, Conceptualization.

## Footnotes

1
http://www.phenoscanner.medschl.cam.ac.uk/
